# Collaborative Development of a Self-Tracking Assisted Psychotherapy Treatment Concept for Refugees With Complex Posttraumatic Stress Disorder: Participatory Action Research

**DOI:** 10.2196/66663

**Published:** 2025-10-07

**Authors:** Lisa Groenberg Riisager, Jakob Eg Larsen, Lotte Huniche, Thomas Blomseth Christiansen, Stine Bjerrum Moeller

**Affiliations:** 1 Department of Psychology University of Southern Denmark Odense M Denmark; 2 Department of Multidisciplinary Trauma Treatment Mental Health Services, Region of Southern Denmark Middelfart Denmark; 3 Department of Applied Mathemathics and Computer Science Technical University of Denmark Kongens Lyngby Denmark; 4 Konsulent Blomseth Hjoerring Denmark

**Keywords:** wearables, mHealth, mobile health, mobile app, digital mental health, self-tracking, One Button Tracker, psychotherapy treatment, mental health, digital technology, digital intervention, mobile phone

## Abstract

**Background:**

Refugees are at high risk of severe mental health challenges due to exposure to war, torture, genocide, and childhood abuse. These experiences may lead to complex posttraumatic stress disorder (CPTSD), a condition that traditional treatments such as cognitive behavioral therapy and eye movement desensitization and reprocessing often struggle to treat adequately. Cultural complexity, limited relevance of standard interventions, and low adherence to therapeutic homework pose additional challenges. Self-tracking technologies offer a promising path for personalized mental health support in patients’ everyday lives, but their integration into psychotherapy remains underexplored.

**Objective:**

This study aimed to collaboratively develop a psychotherapeutic treatment concept for refugees with CPTSD by integrating a personalized, wearable self-tracking instrument, the One Button Tracker (OBT), into psychotherapy. The OBT allows patients to track subjective experiences in the moment they occur, offering a way to bridge therapy sessions and everyday life.

**Methods:**

This study was conducted at a Danish trauma clinic specializing in treatment for refugees and veterans with posttraumatic stress disorder and CPTSD. A Participatory Action Research approach situated within the qualitative paradigm guided the process from November 2022 to April 2024. The codevelopment of the treatment concept involved therapists, patients, clinical psychology researchers, and human-computer interaction researchers (n=21). Qualitative data were gathered through patient interviews, therapist logbooks, and peer supervision sessions, and supplemented by self-tracking data from the OBT.

**Results:**

Across 17 months, the team conducted 40 peer supervision sessions, 2 collaborative workshops, and 25 interviews with 9 patients who participated in therapy for 8 to 24 sessions. Self-tracking durations ranged from 22 to 366 days, covering 1 to 14 target phenomena per patient. The OBT was found to enhance patient engagement by supporting active symptom monitoring and reinforcing therapeutic interventions outside sessions. Therapists reported that the self-tracking data provided valuable insights into patients’ lived experiences, supporting more personalized and context-sensitive interventions. The flexible use of the OBT also allowed patients to shift their focus from distressing symptoms to alternative coping strategies. Furthermore, the integration of self-tracking data strengthened the therapeutic alliance by improving communication and collaboration between patients and therapists. Some technical limitations affected data collection but did not substantially hinder the therapeutic process.

**Conclusions:**

This is the first study to use a Participatory Action Research approach to codevelop a psychotherapeutic treatment concept integrating self-tracking technology for refugees with CPTSD. Findings indicate that the OBT may improve patient engagement, increase adherence to therapeutic tasks, and strengthen the therapeutic alliance. The treatment concept shows promise as a transtheoretical and transdiagnostic approach, offering a flexible and personalized model for psychotherapy. Future research should refine the concept and examine its applicability across broader clinical contexts and populations.

## Introduction

### Background

As of 2023, the global refugee population has reached 30.5 million [1], with many refugees facing severe mental health challenges due to traumas such as war, torture, and childhood abuse [2,3]. These forms of repeated interpersonal trauma can precipitate the development of complex posttraumatic stress disorder (CPTSD) [4,5], a diagnosis recently included in the ICD-11 (International Statistical Classification of Diseases, Eleventh Revision). CPTSD extends the diagnostic framework of posttraumatic stress disorder (PTSD) by including disturbances in affect regulation, self-concept, and interpersonal relationships [3,4,6].

Although established psychotherapeutic approaches such as cognitive behavioral therapy (CBT), eye movement desensitization and reprocessing, and exposure therapy have shown effectiveness in treating CPTSD [7], their implementation is often inadequate in refugee populations. Much of the existing literature on psychotherapy for refugees focuses on PTSD [5], with findings often generalized to CPTSD without accounting for its distinct symptom profile and treatment needs. In particular, trauma-focused approaches may overwhelm patients when introduced prematurely, especially in the context of ongoing stressors, unstable living conditions, and complex grief [4]. Such mismatches often weaken therapeutic engagement [8,9], pointing to the need for treatment approaches that are specifically tailored to the lived realities of refugees with CPTSD.

Mental health professionals working with refugee populations also face specific challenges in addressing trauma. Karageorge et al [10] report that both refugee clients and staff express uncertainty about the value of focusing on traumatic events, with some providers experiencing secondary trauma themselves. Similarly, Peñuela-O’Brien et al [11] found that cultural and language barriers, along with strong emotional reactions from health care professionals, complicate trauma care, necessitating a flexible and person-centered approach.

While specialized treatments such as narrative exposure therapy have yielded promising outcomes [5,12], conventional psychotherapies often remain session-bound and do not fully engage with the everyday realities of refugee patients. Postmigration and socioeconomic stressors, as well as cross-cultural challenges, compound these challenges and can undermine patients’ capacity to engage with therapy in daily life [13]. With between 16% and 38% of refugees meeting the criteria for CPTSD [4], there is a need for treatment models that are closely attuned to the complexity of patients’ everyday contexts and the cultural dimensions of their trauma.

### Personalized Approach to Self-Tracking

Digital mental health interventions increasingly aim to extend therapeutic support into patients’ everyday lives. Among these, self-tracking technologies offer a means of collecting real-time data on thoughts, emotions, and behaviors, thereby enabling interventions that are more closely attuned to individual patterns and contexts [14]. While digital self-tracking draws on the long-standing clinical practice of self-monitoring, where patients record relevant experiences as part of therapeutic homework [15,16], its digital form transforms this activity into a continuous and integrated part of daily life [17].

Unlike passive sensing, which relies on device sensors to collect behavioral data without user input, active self-tracking requires patients to deliberately register their experiences [18,19]. This active engagement can increase awareness and agency, as patients learn to recognize symptom triggers and evaluate the effects of coping strategies between sessions [20,21]. Such data can serve as a concrete reference point in therapy sessions, offering therapists valuable insight into the patient’s daily experiences that would otherwise remain inaccessible [16,22].

For therapists, the integration of self-tracking data can support more targeted, personalized interventions. Instead of relying solely on retrospective self-report or standardized questionnaires, therapists can engage with data that reflect lived experiences as they unfold [19]. Shared interpretation of these data in therapy sessions may also enhance collaboration and therapeutic alliance [18,23].

Despite its potential, research on self-tracking in psychotherapy remains limited. Existing studies have largely focused on smartphone apps or web-based interventions [24], with minimal exploration of how self-tracking might support face-to-face or blended care models. This gap is particularly striking concerning refugee populations affected by trauma. To date, no studies beyond our case study [20] and feasibility pilot study [17] have examined how self-tracking might be adapted to support psychotherapeutic treatment for refugees with CPTSD. Given the complex symptomatology and disrupted daily functioning often seen in this population, exploring self-tracking as a therapeutic tool in this context is both timely and necessary.

### The One Button Tracker and Self-Tracking

To explore the integration of self-tracking in psychotherapy, this study used a research prototype of the One Button Tracker (OBT), a novel wearable self-tracking instrument that enables in-the-moment registration of a self-chosen subjectively experienced phenomenon relevant to the patient’s mental health challenges [17,20,25]. The OBT is designed with a single-button interface that enables patients to record subjective experiences with minimal effort or disruption to daily life [17,20,25-27]. This design ensures that self-tracking becomes a seamless part of the patient’s daily routine, reinforcing engagement with the therapeutic process.

A central aspect of the OBT’s application is its personalization. Therapists and patients collaboratively decide which specific phenomenon to track between sessions, tailoring the self-tracking practice to each patient’s lived experience and current therapeutic focus [17,20]. This user-defined input differentiates the OBT from conventional symptom checklists or mobile apps that often offer fixed categories. As a form of real-time, patient-driven data collection, the OBT functions both as therapeutic homework and as an instrument for bridging experiences between therapy sessions [17]. It enables patients to become more aware of thoughts, emotional responses, and behavioral patterns as they unfold, while providing therapists with contextualized data that can inform and personalize therapeutic interventions [28].

The OBT is positioned within the methodological framework of ecological momentary assessment [29,30], allowing for in-the-moment data capture of naturally occurring phenomena. These data are visualized through a purpose-built interface and used during sessions to support reflection, pattern recognition, and collaborative decision-making between therapist and patient [17,20].

### Participatory Action Research

Given the novelty of integrating wearable self-tracking technology into psychotherapy, especially within a culturally complex and clinically vulnerable population, a preliminary feasibility pilot study was conducted with approval from the Regional Committees on Health Research Ethics for Southern Denmark [17]. The study involved 2 patients and 2 therapists and focused primarily on the usability and integration of the OBT and the associated visualization tool into psychotherapeutic practice. Rather than assessing treatment outcomes, the aim was to explore initial engagement, practical feasibility, and interdisciplinary collaboration between human-computer interaction (HCI) specialists and clinical psychologists [17].

Building on these findings, the current study used a Participatory Action Research (PAR) design to codevelop a treatment concept in close collaboration with therapists, patients, and researchers. Through iterative cycles of planning, action, and reflection, the PAR approach allowed for continuous adaptation of the treatment method in response to real-world clinical challenges and patient feedback. This methodology supported the development of a psychotherapeutic approach that is both culturally responsive and grounded in the lived experiences of refugees with CPTSD [31-34].

### Aim of This Study

This study aims to codevelop a psychotherapeutic treatment concept that integrates wearable self-tracking and the data visualization tool to personalize interventions for refugees with CPTSD. By focusing on the phenomenological and cultural experiences of individual patients, this approach seeks to bridge the gap between therapy sessions and the challenges refugees face in their daily lives, creating a more data-informed and personalized therapeutic approach.

## Methods

### Clinical Setting

This study was conducted at the Department of Multidisciplinary Trauma Treatment (DMTT) within the Mental Health Services in the Region of Southern Denmark. DMTT specializes in treating refugees and veterans enduring PTSD and CPTSD, following experiences from war, political persecution, and torture. The clinic uses a multidisciplinary treatment approach, where a team consisting of psychologists, psychiatrists, physiotherapists, nurses, and social workers collaboratively assesses and tailors treatment to each patient. Psychotherapy is conducted by psychologists and is primarily based on CBT and narrative exposure therapy. Psychologists receive monthly supervision. Treatment duration at DMTT is flexible, considering the patient’s psychological, social, and somatic complexities [20]. If needed, patients have access to virtual interpreter services during sessions via an iPad (Apple Inc) app.

### Design

This study, conducted from November 2022 to April 2024, used a PAR design. Central to the PAR process were iterative cycles of reflection and evaluation, driven by the experiences of both therapists and patients using the self-tracking instrument and data visualization tool within the therapy setting [35].

Peer supervision sessions were an integral part of both the iterative PAR process as well as the data collection. These sessions involved researchers and therapists collaborating to discuss the use of self-tracking technology in therapy. The agenda for each session was based on therapists’ logbooks on the patient’s therapy session, which documented their observations and experiences in applying the OBT and the data visualization tool. Through qualitative reports from these meetings, peer supervision sessions contributed to the development of the treatment concept.

To ensure transparency and quality in both design and reporting, we drew on the best practice principles for PAR as outlined by Smith et al [36]. These principles emphasize attention to power dynamics, iterative co-construction, and the inclusion of diverse stakeholder perspectives. Rather than following a conventional reporting checklist, this approach allows for a nuanced representation of the collaborative and evolving nature of PAR in a clinical context [36].

Given the focus on trauma within a vulnerable population, we engaged patients as indirect collaborators [37]. Their involvement was captured through interviews at the beginning, middle, and end of treatment, ensuring that their perspectives shaped the research process [38]. These patient insights not only informed the ongoing refinement of both the technology and treatment concept but also enhanced peer supervision discussions, helping therapists improve their use of the technology during therapy sessions.

In this study, qualitative data from interviews and observations were prioritized to capture participant experiences, while the self-tracking data played a descriptive role. This study’s framework was defined by two key components: (1) the self-tracking instrument and data visualization tool, and (2) a preliminary model of the treatment concept as introduced in the pilot study.

### Ethical Considerations

This study was approved by the Regional Committees on Health Research Ethics for Southern Denmark (ID: S-20210019 CSF) and conducted per the Danish Data Protection Act and the EU General Data Protection Regulation. Before participating in this study, patients provided both oral and written informed consent, while therapists gave oral consent. All participants received written and oral information about this study and provided informed consent before participation. The consent process made clear that data would be anonymized and used for research purposes only within the scope of this approved study. No additional consent was required for analysis, as the original consent covered both data collection and subsequent analysis. To protect participants’ privacy and confidentiality, all data were anonymized at the point of collection and securely stored on institutional servers in line with the University of Southern Denmark’s data management policies. Only authorized research team members had access to the data. Participants were not compensated for their involvement in this study. No identifiable individuals are depicted in any figures or supplementary materials.

### Self-Tracking Instrument and Data Visualization Tool

**Figure 1:**
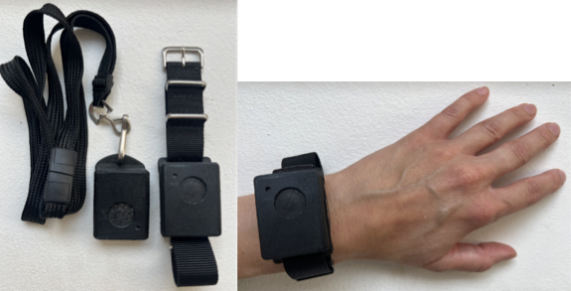
The One Button Tracker instrument shown in two different configurations: (1) worn with a neck strap and (2) worn with a wrist band.

The OBT is a wearable self-tracking instrument, a research prototype specifically designed for quick, in-the-moment registration of a subjectively experienced phenomenon (Figure 1). Equipped with a single button to enhance user-friendliness, the OBT prototype measures 41×31×12.5 mm, offering versatile wearability options, around the neck, in the pocket, or on the wrist, adjusting to individual preferences [17,20]. When the button on the OBT is pressed, it responds with vibrotactile feedback that matches the duration of the press, serving as a confirmation that the press and its length have been recorded. The OBT’s internal storage logs the temporal data associated with each input event, including both the timestamp and duration of the button press. This design emphasizes user adaptability and discretion, minimizing intrusion while facilitating the collection of data of high temporal resolution. It can be operated without visual attention.

**Figure 2:**
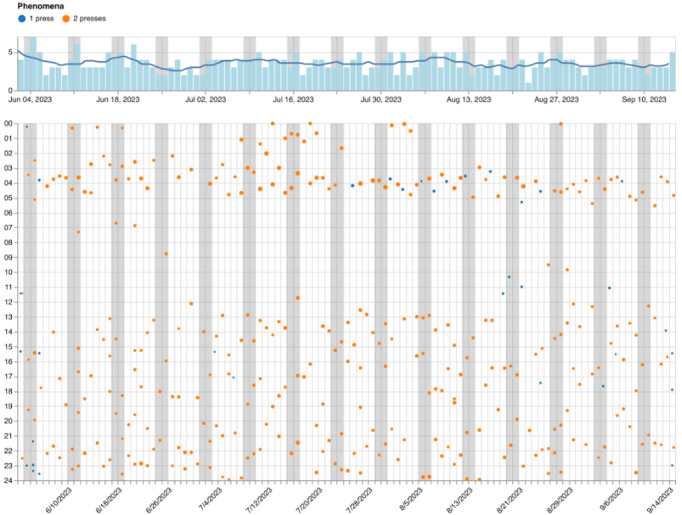
A screenshot of the web-based calendar visualization tool displaying self-tracking data from patient P7. The bar chart at the top shows the total number of observations per day, while the line represents a 1-week moving average. The scatter plot depicts the first 105 of 252 days of observations (x-axis) and the times of the day the observations were made (y-axis). Each dot represents a single observation made. P: patient.

The patient can choose to track 1 or 2 phenomena with the OBT, differentiated by single or double button presses, respectively. This is referred to as either a 1- or 2-press protocol. The tracking of subjectively experienced phenomena with the OBT is visualized with the data visualization tool. This involves the patient handing the OBT to the therapist, who then connects it to a desktop or laptop computer using a USB cable. This enables access to a specialized web-based visualization tool that provides a detailed, calendar-like overview of the data, as shown in Figure 2. It highlights the frequency and distribution of identified phenomena across different times of day and days, enhancing a data-driven collaborative analysis between therapist and patient [20].

### Self-Tracking Assisted Psychotherapy Treatment Concept

**Figure 3:**
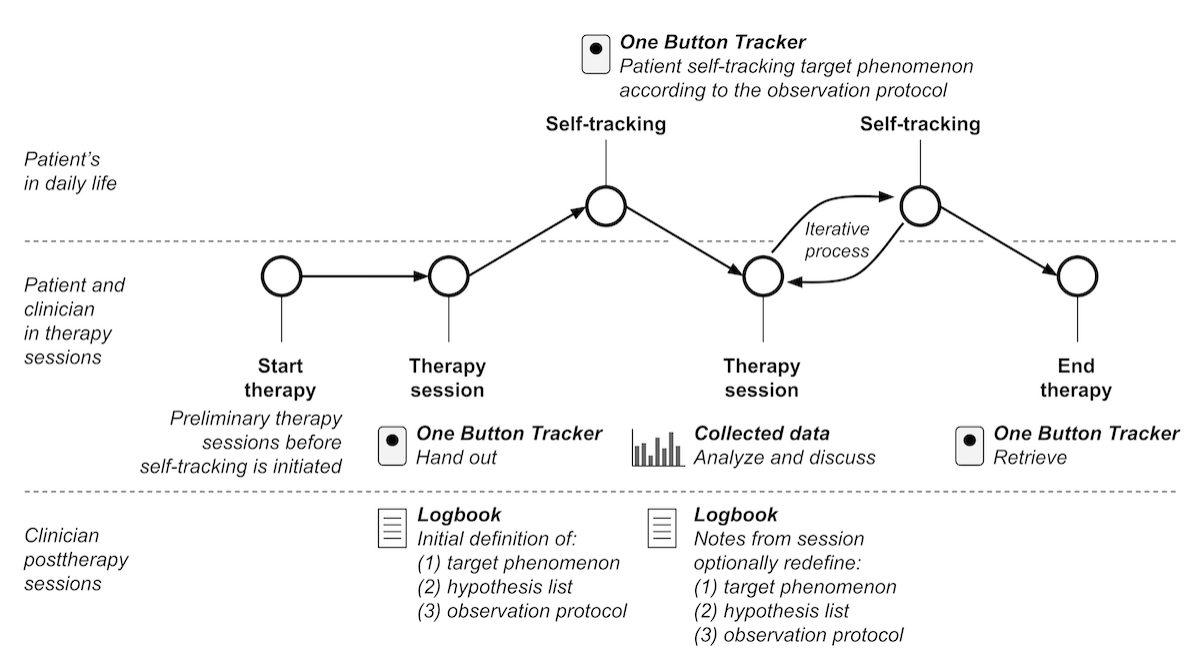
Flowchart of the self-tracking-assisted psychotherapy process in the treatment concept. The approach involves the patient self-tracking a target phenomenon in daily life using the OBT instrument. Following the initial definition of the observation protocol, subsequent therapy sessions incorporate collaborative analysis and discussion of collected data by the patient and therapist. This informs the psychotherapeutic process and guides clinical interventions through an iterative process, allowing for redefinition of target phenomena and observation protocols as needed.

Drawing on the principles of the Quantified Self community [39], the OBT is conceptualized within psychotherapeutic treatment as a digital personal diary to track the occurrences of a subjectively meaningful phenomenon. A core aspect of this treatment approach is its focus on personalization, where patients actively choose what they wish to track based on their unique mental health challenges and therapeutic goals. The process of choosing a relevant phenomenon to track is introduced to the patient during the initial therapy sessions, where the rationale for using the OBT is thoroughly explained. An overview of the self-tracking-assisted psychotherapeutic process is presented in Figure 3, with key concepts detailed in Table 1.

**Table 1:** Key concepts in the self-tracking-assisted psychotherapy treatment concept.

Key concept	Description
Target phenomenon	A distinct experience, such as a trigger, symptom, or behavior relevant to the patient’s mental health challenges.
Hypothesis list	The patient’s and the therapist’s joint assumptions about the expected distribution of the occurrences of the target phenomenon and potential triggers.
Observation protocol	An agreement on how and when the patient presses the button on the OBT^a^ and how to wear the OBT.
Evaluation of observation protocol	Assessing the tracking period between sessions, as well as the patient’s ability to identify the target phenomenon, and the patient’s experience with tracking.

^a^OBT: One Button Tracker.

In therapy sessions, patients and therapists collaborate to identify a subjectively significant target phenomenon that the patient chooses to track between sessions. Unlike traditional methods where tracking parameters are predefined through validated questionnaires or the therapist, this approach empowers patients to decide what is most relevant to them, fostering a more personalized and engaged tracking process. An observation protocol is collaboratively defined, outlining markers of the chosen phenomenon, whether somatic, mental, or situational, to ensure that patients can accurately recognize and track their selected target phenomenon.

To further align with patient expectations regarding the occurrence of target phenomena, a hypothesis list is developed. This list, reflecting the patient’s interpretations of their experiences, is subsequently explored in the next session to examine the implications and insights derived from these hypotheses. As self-tracking progresses, each therapy session includes an evaluation of the observation protocol*,* ensuring that the tracking activity remains clinically focused and relevant to the patient’s treatment goals. This iterative process is central to maintaining the personal relevance and clinical utility of the tracking activity, with in-session analysis of the data guiding any necessary adjustments.

Throughout therapy, this dynamic approach supports the continuous refinement of the target phenomena being assessed. The therapy sessions serve as a space where the tracking process is regularly revisited and updated as needed, always with the patient’s input at the forefront. This ensures that the self-tracking activity not only aligns with but actively supports the evolving goals of the treatment.

### Participants and Recruitment Procedure

This study involved 21 participants across 4 key groups: therapists, patients, clinical psychology researchers, and HCI researchers. First author LGR was the principal investigator of this study. Initially, 8 therapists from DMTT participated in this study, having been selected for their interest in the project. Over time, this number was reduced to 4 due to resignations and other commitments in the clinic. Additionally, the first author, LGR, who is both a clinical psychology researcher and a trained psychologist, also participated as a therapist in this study. On the research and development side, 2 clinical psychology researchers (the first author, LGR, included) and 2 HCI researchers participated in this study.

On the patient side, 9 patients from DMTT were included through convenience sampling, based on the following eligibility criteria: being referred for psychiatric treatment, having refugee status, being aged 18 years or older, having a diagnosis of CPTSD according to the International Trauma Interview (Roberts N et al, unpublished data, 2019), and being proficient in Danish.

### Data Collection

This study included both qualitative and quantitative data. The data collected included (1) therapy session attendance (ie, the number of therapy sessions patients attended while using the OBT); (2) patient interviews conducted before, during, and after treatment to explore patients’ experiences with using the OBT and collaboratively analyze self-tracking data in sessions; (3) self-tracking data collected from the OBT to explore patient engagement and data patterns; (4) therapists’ logbooks for each patient with observation protocols and accounts of interventions used in the therapy session to document the therapeutic process; (5) peer supervision meeting minutes with reflections on therapeutic strategies and adjustments discussed during supervision sessions; and (6) workshops (ie, contributions to the collaborative development of the treatment concept).

All interviews were carried out by the first author, LGR, except interviews with 1 patient for whom LGR acted as therapist. These interviews were conducted by coauthor SBM.

### Research Process

**Figure 4:**
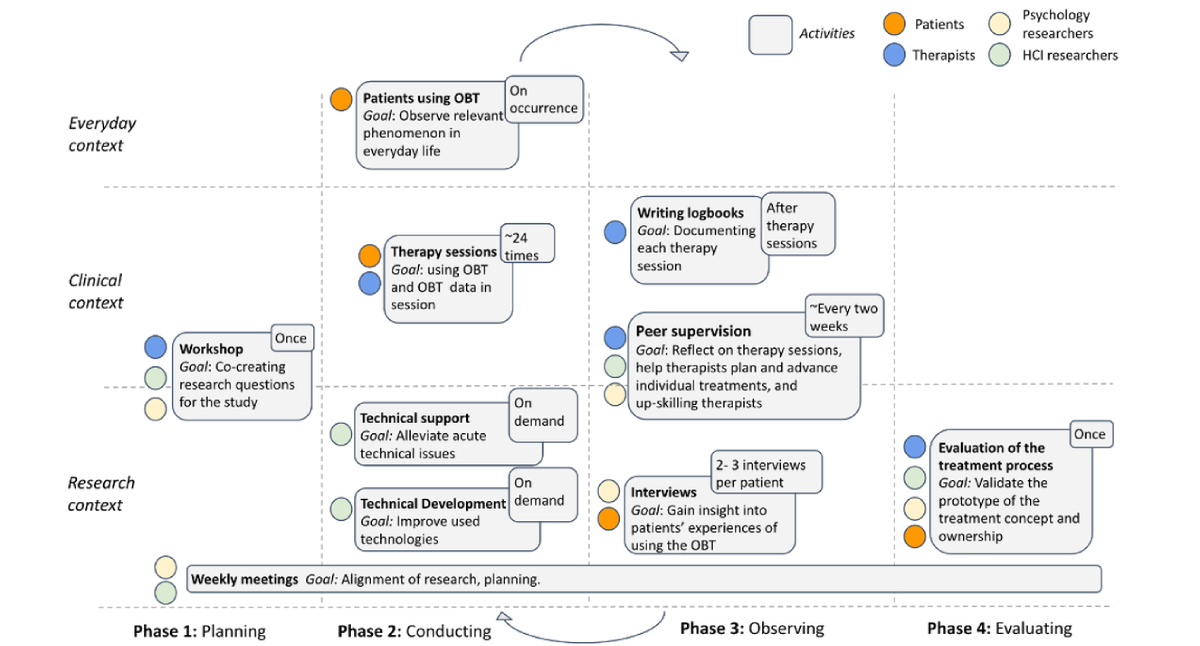
Overview of the phases, participants, and activities of the PAR research process. HCI: human-computer interaction; OBT: One Button Tracker; PAR: participatory action research.

This study was conducted from November 2022 to April 2024. The phases of the PAR process in this study are visualized in Figure 4. Phases and corresponding activities are described in the following.

### Phase 1: Planning

In November 2022, before patient enrollment, a 4-hour workshop was held to familiarize therapists with the research design, share findings from the pilot study, and collaboratively establish the objectives and research questions (RQs) of this study. Nine participants participated in the workshop, which included 5 therapists, 2 HCI researchers, and 2 clinical psychology researchers.

To begin the group discussion, therapists were introduced to the area of interest, specifically the role of the self-tracking instrument and data visualization tool in linking patients’ daily lives with therapy sessions. They were then divided into 3 groups and spent 60 minutes discussing the use of the technology in psychotherapy. Discussions were audio-recorded, and themes were written on A1-sized posters. Group presentations subsequently synthesized these discussions into a poster with the final research themes and questions:

RQ1: ethical and practical considerations: How do therapists navigate ethical concerns when integrating the OBT into psychotherapy, and what practices ensure the protection of patient well-being?

RQ2: patient experiences with using the OBT: How do patients describe their experiences with the OBT in therapy, particularly regarding their personal engagement and self-awareness?

RQ3: setting and achieving therapeutic goals: How do therapists and patients collaboratively determine and reflect on the goals for using the OBT in therapy, especially concerning trauma processing and emotional regulation?

RQ4: interpretation and use of self-tracking data: How do therapists interpret and integrate self-tracking data into therapy sessions, and how does this affect the therapeutic process?

RQs 1, 3, and 4 will be addressed in this paper, while RQ 2 will be addressed in a separate paper.

### Phase 2: Conducting

Phases 2 and 3 were conducted iteratively from November 2022 to April 2024. In this phase, therapists integrated the use of the OBT and the data visualization tool into therapy, following the preliminary model of the treatment concept from the pilot study. Therapists decided when it was appropriate to introduce the OBT in treatment. They collaborated with patients in selecting a target phenomenon, and patients were then guided by therapists to integrate the use of the OBT into their daily lives. HCI researchers provided a worksheet to support therapists in this process (Multimedia Appendix 1).

Weekly meetings with the principal investigator and HCI researchers reviewed the use of the self-tracking instrument and data visualization tool in therapy, addressed technical issues, and discussed methodological insights. Throughout this study’s period, HCI researchers offered technical support and made iterative improvements to the data visualization tool, based on feedback acquired and user needs identified, especially during the peer-supervision sessions.

### Phase 3: Observing

After each session, therapists recorded key information in logbooks as part of the data collection. In the logbook, therapists outlined the key concepts of the therapy session, a brief statement on the therapy session, whether the self-tracking activity had led to insights for the patients, and any questions that might have come up on the use of the self-tracking technology. The information and questions from these logbooks were then used to outline the agenda for peer supervision sessions, ensuring that key reflections and issues were addressed. During peer supervision, therapists shared progress and challenges, which helped them enhance their skills in using self-tracking data to inform therapy. Discussions in these sessions focused on interventions, observation protocols, and session reflections.

In this phase, patient interviews served dual purposes: they provided insights into patient experiences and enabled patients to be included as cocreators. Insights from these interviews were shared with therapists during peer supervision sessions, contributing to the cocreation of the preliminary treatment concept.

### Phase 4: Evaluating

This phase involved a 90-minute hybrid workshop with 4 remaining therapists, 2 HCI researchers, and the first author, LGR, to evaluate the findings from phase 3. The purpose was to evaluate the findings from phase 3 by collaboratively reviewing and refining the treatment concept based on preliminary, thematically organized data.

A web-based shared whiteboard facilitated real-time collaboration, allowing participants to categorize ideas and reflections into distinct themes. This collaborative evaluation process helped streamline and clarify key aspects of the treatment concept. The themes identified during the workshop included (1) introduction and start, (2) integration and engagement with the OBT, (3) communication and relationship building through data and the OBT, (4) therapeutic application and adaptation, (5) data utilization and ownership, and (6) patient agency and autonomy.

The analysis of these themes is presented in the Results section.

### Data Analysis

Quantitative self-tracking data and qualitative data from workshops, logbooks, peer supervision sessions, and interviews were included in the collaborative development of the treatment concept.

For this paper, quantitative data provided by the OBT instruments were processed. Interactions with the instruments were categorized as single or double presses. A double press was defined as a second press occurring within 1500 milliseconds of the first; otherwise, it was defined as a single press. The total number of single and double presses was calculated, along with the average number of observations per day and per hour.

The qualitative data were analyzed by the first author, LGR, under the supervision of the coauthors, using the 6 phases of reflexive thematic analysis by Braun and Clarke [40]: familiarization with the data, coding, generating initial themes, reviewing themes, defining and naming themes, and reporting the findings. NVivo (version 12; Lumivero) software was used to assist in this analysis.

## Results

### Overview

Over 17 months, we conducted 40 peer supervision sessions, 2 workshops, and 25 patient interviews involving 9 patients whose treatment durations varied from 4 to 24 sessions. Most patients were female (6/9, 67%), with ages ranging from 22 to 63 years, resulting in a mean age of 42.8 years.

### Self-Tracking Data

Table 2 presents an overview of the number of protocols, target phenomena, treatment duration, and self-tracking data for the 9 patients. The duration of OBT use varied, ranging from 22 to 366 days, with the total number of observations spanning from 37 to 4733, resulting in an average daily number of observations from 0.7 to 37.0. Patients tracked between 1 and 14 different target phenomena, equating to between 1 and 17 distinct observation protocols.

**Table 2:** Summary of patients in this study, detailing demographic information, treatment sessions, and self-tracking instrument usage. Days without data indicate periods where no data was collected due to technical issues, lost instrument, or lack of instrument access.

Participant	Gender	Age (years)	Total sessions with tracking	Total days with tracking	Days with data	Days without data	Number of observations	Daily average observations	Protocols used	Target phenomena tracked
P1^a^	F^b^	31	12	92	62	30	1276	20.6	4	3
P2	M^c^	63	17	112	87	25	347	4.0	4	4
P3	F	59	24	324	289	35	2038	7.1	17	14
P4	F	22	22	262	185	77	130	0.7	10	8
P5	F	32	13	366	278	88	980	3.5	8	8
P6	M	57	12	246	128	118	4733	37.0	5	5
P7	F	52	20	252	212	40	708	3.3	5	4
P8	M	47	8	175	58	117	188	3.2	1	1
P9	F	22	4	22	22	0	37	1.7	3	3

^a^P: patient.

^b^F: female.

^c^M: male.

Eight out of 9 patients had days without observations during the part of their treatment where the OBT was in use. These gaps were primarily due to technical issues (eg, battery problems), instances where the OBT was misplaced, or situations where the therapist forgot to return the tracking instrument at the end of a therapy session. In some cases, patients actively chose not to track on specific days, such as when they were on vacation (and therefore also wanted to take a break from their treatment) or when they had visitors whom the patients did not want knowing about their treatment.

**Figure 5:**
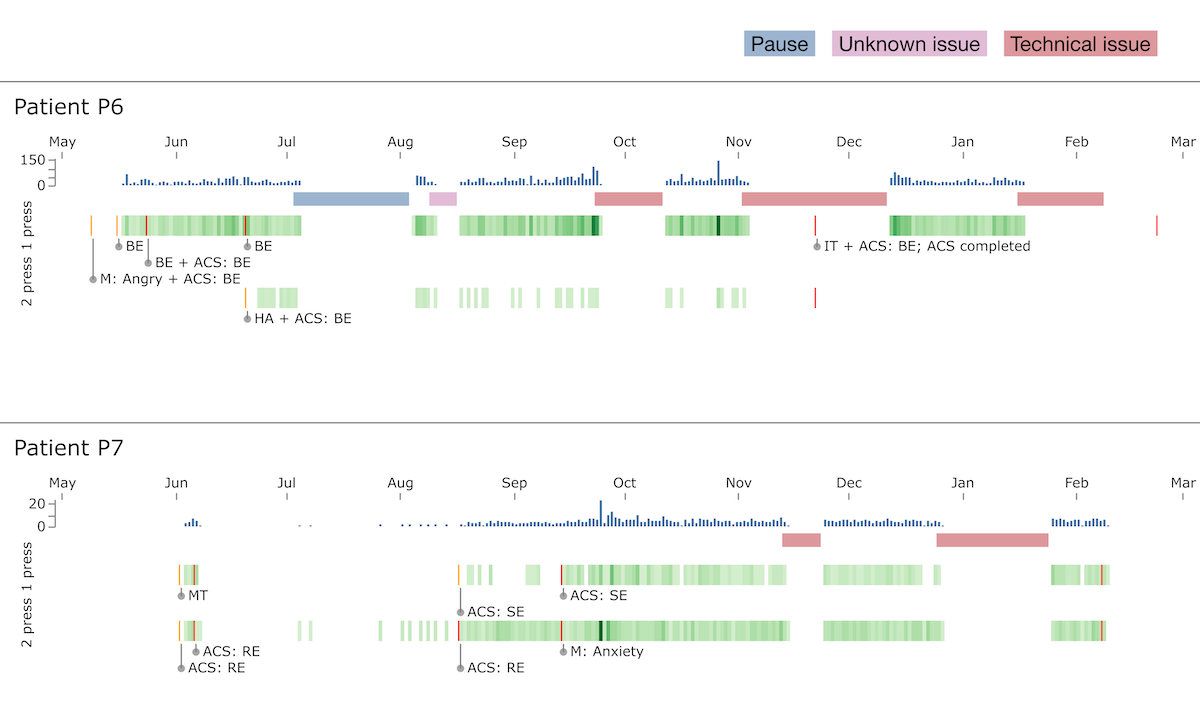
Daily observations, outages, and observation protocols for patients P6 (male, 56 years) and P7 (female, 52 years) from May to March the following year. The topmost row in each patient section displays the histogram of total daily observations, highlighting the frequency and distribution of data collection over time. Below the histograms, horizontal bars denote periods of outages due to technical issues, process changes, or adherence lapses, with different colors representing distinct types of interruptions. The subsequent heat maps illustrate the occurrence of specific phenomena, with the first heat map indicating phenomena that required a single press for recording and the second for those requiring 2 presses. Key changes in observation protocol and the types of phenomena (eg, BE for breathing exercise and ACS for alternative coping strategy) are annotated within the heat maps. ACS: alternative coping strategy; BE: breathing exercise; HA: high arousal; IT: intrusive thoughts; M: monitoring; MT: monitoring trigger; P: patient; RE: recorded exercise; SE: somatic empowerment.

Figure 5 illustrates the self-tracking data of 2 of the 9 patients (P6 and P7), selected to exemplify the variations in daily observations using 1-press and 2-press protocols. During their treatment spanning 8-9 months, the patients each tracked 2 distinct phenomena. In the monitoring category, these phenomena were a specific intrusive thought (IT) and a trigger of a problematic behavior, respectively. In the intervention category, an alternative coping strategy (ACS) and recording of a completed therapeutic exercise, respectively, were tracked. During this period, the phenomena of interest were adjusted multiple times to better align with their evolving therapeutic needs.

The data visualization shows a timeline of the tracked phenomena for both P6 (male, 57 years) and P7 (female, 52 years), using specific abbreviations. For example, phenomena such as breathing exercise (BE), ACS, high arousal, ITs, somatic empowerment, recorded exercise, monitoring trigger, and monitoring are shown. By analyzing these self-tracking activities, we can see how the patients engaged with the interventions and adapted their tracking practices based on their changing therapeutic goals throughout treatment.

### Key Findings

**Figure 6:**
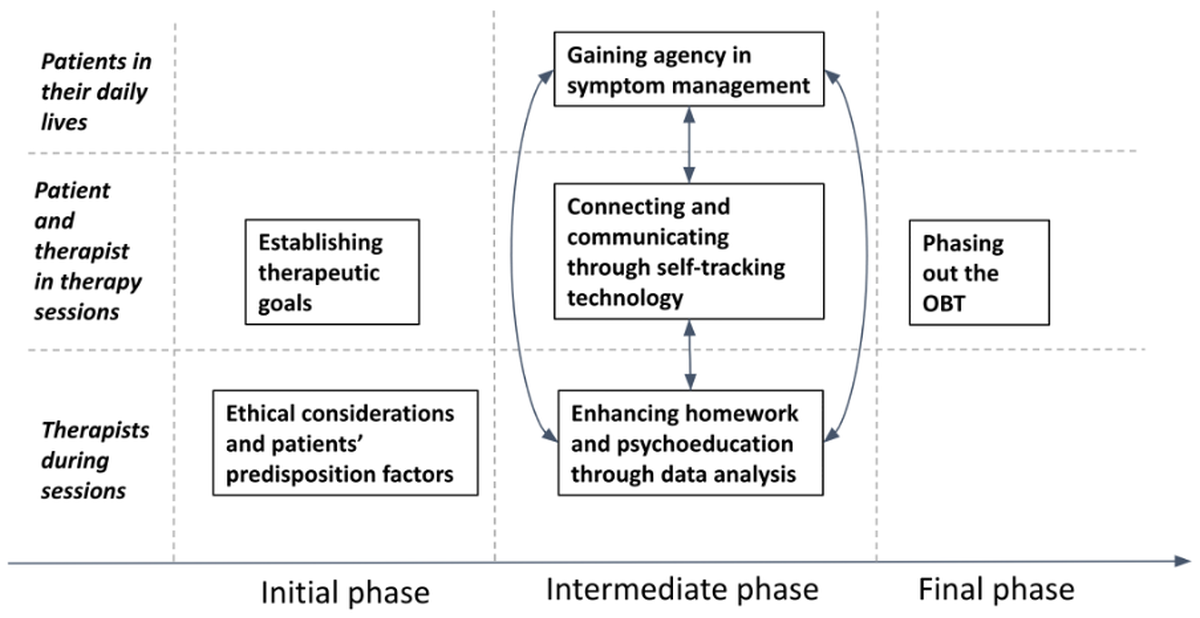
A timeline illustrating key thematic findings across 3 phases of the treatment, highlighting the clinical utility of the technology at each therapeutic stage. OBT: One Button Tracker.

The qualitative findings are presented along a timeline (Figure 6), structured around 3 distinct phases of the therapeutic process. As the context was found to shape how the OBT was used and experienced, the timeline is organized by phase, highlighting both the primary actor and the setting involved. This structure ensures that each theme is grounded in its specific context, offering a clear and coherent view of how the treatment unfolded over time.

### Initial Phase

#### Ethical Considerations and Patients’ Predisposition Factors

Our final workshop highlighted that evaluating patients’ predisposition factors was essential during the initial phase of integrating the OBT into therapy. This preliminary assessment enabled therapists to gauge each patient’s capacity to manage the mental load associated with self-tracking. For instance, 2 patients declined participation, explaining that attending regular therapy sessions was already overwhelming, and the added strain of using the OBT would be too burdensome.

Our findings indicate that self-tracking technology is most relevant in psychotherapy for patients who already exhibit a motivation for self-help. In interviews, several patients described how beginning therapy and using the OBT encouraged them to take a more active role in managing their mental health. This impression was echoed in peer supervision, where therapists observed that the OBT appeared to cultivate a sense of responsibility, leading to greater patient engagement in the therapeutic process.

Logbook entries emphasized the importance of screening for depressive symptoms before introducing the OBT. In depressive states, patients often gravitate toward negative emotions or experiences, which can distort the focus of the self-tracking activity and diminish its therapeutic value. A drop in engagement or sparse data may signal that the patient is overwhelmed and should be addressed by the therapist. At the same time, these patterns make the OBT particularly valuable: by exposing subtle shifts in mood and behavior, it helps identify when patients are struggling.

For example, after 6 sessions, 1 patient showed signs of increased withdrawal, reflected in a noticeable drop in OBT interactions. By analyzing the self-tracking data, the therapist identified prolonged social isolation as a contributing factor to worsening depressive symptoms. In response, the therapist introduced psychoeducation and cognitive restructuring and repurposed the OBT to track positive thoughts about relatives and encourage social interactions.

At the same time, some concerns arose about the OBT’s suitability for patients experiencing emotional instability or complex relational dynamics. One patient, who frequently cancelled sessions and exhibited overall emotional instability, found the OBT overwhelming, particularly when compounded by other life challenges. After 8 sessions, she and the therapist agreed to discontinue its use. Still, she reflected that the brief period of self-tracking had offered valuable insights into her maladaptive patterns, suggesting that it can still offer therapeutic benefits even when used briefly.

#### Establishing Therapeutic Goals

In peer supervision sessions, it became evident that the initial phase of therapy served as an exploratory period where therapists and patients collaboratively worked to identify and define target phenomena that aligned with the patient’s broader treatment goals. This phase was essential for assessing whether tracking a specific phenomenon could reveal meaningful patterns or contextual insights into the patient’s mental health challenges. Therapists emphasized the importance of choosing phenomena that patients could easily recognize and reliably track in everyday life. These were typically introduced and refined over 2 sessions, with attention to identifying precise mental or somatic markers to ensure relevance and accuracy.

Although therapists and patients agreed that self-tracking should serve treatment goals, logbook data showed that maintaining this alignment was difficult in practice. While some target phenomena were clearly defined at the outset, their connection to the overarching goals sometimes weakened over time, particularly when the target phenomena or observation protocols were frequently adjusted. Defining phenomena with sufficient precision also remained a recurring difficulty. Both patients and therapists sometimes relied on abstract or vague terms such as “anxiety” or “anger,” which lacked the specificity needed for self-tracking to meaningfully inform therapeutic work. As a result, therapists found it difficult to work with stable, goal-aligned data throughout treatment.

This difficulty was less pronounced when the target phenomena involved concrete coping strategies, such as BEs or visualization techniques. Tracking these interventions provided a more structured and consistent therapeutic focus, likely because therapists were already familiar with these well-established interventions. This familiarity made it easier to guide patients in tracking their use, thereby maintaining a clearer link between the tracking activities and treatment goals. In contrast, identifying target phenomena that were personally relevant to patients, such as specific thoughts, emotions, or behaviors, was new to both therapists and patients. This novelty may have contributed to greater challenges in maintaining a clear therapeutic focus, as it required therapists to support patients in identifying and working with subjective experiences that were more diffuse and harder to define.

However, when tracking protocols that focused on alternative coping strategies without a clearly defined underlying problem, a disconnect sometimes emerged between the therapist and patient. As seen in logbooks, in these cases, patients were asked to track strategies such as BEs, but it remained unclear when and why they should be used. Without a shared understanding of the problem that the strategy was meant to address, the tracking activity lost direction and therapeutic relevance. These instances point to the importance of clearly defining both the problem and the specific context in which the coping strategies are to be applied. When this link is established, tracking coping strategies can become a meaningful and targeted part of the therapeutic process rather than a standalone activity.

The use of the OBT can also help bring such misalignment to light. As the use of the instrument requires clear definitions of what is being tracked, it can expose when interventions are being applied without a strong connection to the underlying issue. While the presence of a disconnect cannot be resolved by technology alone, the use of the treatment concept along with the OBT makes it more apparent for the therapist to notice such gaps. In some cases, this increased clarity enabled therapists to re-establish the connection between the problem and the coping strategy.

### Intermediate Phase

#### Gaining Agency in Symptom Management

As therapy progressed, patients described in interviews a dynamic interaction with the OBT, which helped them to gain agency over distressing thoughts and maladaptive behaviors as they occurred. In the intermediate phase, data from interviews, logbooks, and peer supervision sessions indicated that most patients experienced a sense of agency in managing their mental health challenges, even among those who engaged minimally with the OBT. According to interviews, carrying the OBT and pressing the button during everyday activities was often guided by a clear intention and hope for change. For example, notes from peer supervision and logbook entries showed how 1 patient, a young woman, initially tracked binge eating episodes as her target phenomenon but later refined it to track the urge to binge eat instead. Over 4 sessions, she reported in an interview that she gained insights into her behavioral patterns and explained how pressing the button served as a mindful pause that allowed her to reassess her emotional impulses and disrupt her habitual response to emotional discomfort.

Several patients described in interviews that tracking coping strategies felt empowering and offered a way to take constructive action in moments of distress. Many used the OBT to shift attention away from ITs, helping them regain mental presence. A woman of Afghan descent, overwhelmed by catastrophic thoughts related to her health, described an immediate cognitive shift with a button press, comparing it to “turning off a light.” This simple yet impactful action helped her gain agency over her anxiety. In the interview, she described how pressing the button required a deliberate change in mindset, demonstrating how a button press facilitated a cognitive shift for her.

Interviews and peer supervision data further revealed that, for some patients, the vibrotactile feedback from the press developed into an intervention in its own right. One patient, for example, began pressing the button for extended periods—up to 43 seconds—and requested that the vibrotactile feedback be intensified to resemble an electric shock. This sensation, he explained in an interview, helped distract him during moments of intense anger.

In several interviews, patients described the vibrotactile feedback as part of an empowering feedback loop. They emphasized how the OBT’s immediate, physical response allowed them to feel understood without having to explain themselves. One patient remarked that the OBT did not ask “dumb questions,” describing it instead as a silent but supportive companion in everyday life.

#### Engagement and Psychoeducation Through Data Analysis

The iterative nature of the therapeutic process became particularly evident during the intermediate phase, as self-tracking data from the OBT was regularly discussed in sessions. According to therapists in peer supervision, the process initially involved a detailed data analysis, but gradually shifted toward establishing a baseline of patient engagement. One therapist, for example, described how a patient developed an observation protocol where they pressed the button once for anxiety-related tremors and twice for an ACS during the early stages of trauma-focused therapy. Although this phase of trauma-focused therapy typically involves a temporary increase in symptoms, the data showed that the patient’s symptoms did not escalate, offering reassurance to the therapist that the patient was coping well despite expectations of initial worsening.

Logbook entries further underscored the value of collaborative data analysis in identifying distressing contexts. In 1 case, a patient realized through her data that visits to her doctor triggered significant anxiety, which she traced back to the fact that her abusive ex-husband was registered at the same clinic. This insight, discussed during a therapy session, led her to switch doctors to avoid the trigger. Another patient tracked the use of BEs in both calm and stressful situations and, through data analysis with the therapist, identified a link between job-related stress and worsening nightmares. Recognizing this pattern reinforced the importance of maintaining the BE as a coping strategy, helping the patient sustain the practice in daily life.

Data visualization also played a key role in psychoeducation. In interviews, some patients described a sense of satisfaction in seeing visual evidence of their progress. Being able to track improvements over time helped them better understand their mental health challenges and identify which interventions were most effective. One patient of Bosnian descent, for example, reported in an interview that he gamified his data by trying to increase the number of button presses linked to interventions rather than symptoms, which was an approach that symbolized his efforts to take more active control over his recovery.

At the same time, peer supervision sessions revealed several challenges related to the use of self-tracking data. Therapists noted that the level of detail in the visualizations could be difficult to manage in session. Small text sizes and complex graphical elements sometimes made it harder for both therapists and patients to engage meaningfully with the data. In 1 case, a patient became confused when the color scheme of the visualization changed due to a setup error, which disrupted his patient’s connection to the data and led to reduced engagement in subsequent therapy sessions. Interviews also showed that some patients struggled to recall the context of individual data points, likely related to difficulties with autobiographical memory. One therapist described how the sheer density of the data made it difficult to work with in detail during therapy sessions.

#### Connecting and Communicating Through Self-Tracking Technology

Data from the OBT proved instrumental in fostering a shared understanding of symptom patterns, contextual triggers, and patient engagement, which appeared to positively influence the therapeutic alliance. As defined by Bordin [23], the therapeutic alliance consists of agreement on goals, collaboration on tasks, and an emotional bond between patient and therapist. Across interviews and peer supervision sessions, therapists and patients alike described how the integration of self-tracking data appeared to strengthen all 3 components of the alliance.

Engaging with self-tracking data often establishes a sense of partnership between therapists and patients. In interviews, patients emphasized the communicative value of the data, noting that it offered their therapists nonverbal insights into their daily experiences. For some, the data collected and the vibrotactile feedback functioned as a nonverbal connection, reinforcing their sense of being heard and supported, and contributing to an increased feeling of agency. Therapists, in turn, reported that the data helped them gain a clearer picture of when and where symptoms occurred, enabling more targeted interventions. This was particularly valuable when working with patients who were not fluent in Danish, as it reduced the pressure on verbal communication. In an interview, 1 patient explained that he did not have to try to express himself because the therapist could see how he was feeling through the data.

The perceived connection through the OBT offered a sense of continuity between sessions and a source of support during moments of isolation or distress. One woman, for example, described in an interview how the OBT served as a direct link to her therapist. Before using the instrument, she struggled with anxiety and tended to isolate herself as a coping strategy. The presence of the OBT changed this pattern by serving as a tangible reminder of therapy, helping her stay engaged and feel supported between sessions. For her, pressing the button became a way to feel heard without having to speak, a nonverbal affirmation of her therapeutic connection.

Sharing and collaboratively analyzing the data during therapy sessions also appeared to strengthen the therapeutic alliance. According to patient interviews, this process helped patients feel recognized and supported, and ensured that both the therapist and patient remained aligned on the goals and tasks related to the tracking activity. Peer supervision sessions provided additional examples of how the OBT enhanced collaboration in practice. One patient described pressing the OBT button to ensure that their experiences were communicated to the therapist, even without verbal explanation. This perception was reinforced when the therapist reviewed the data in session and invited reflection, creating a shared space of understanding and helping the patient feel seen.

### Final Phase: Phasing Out the OBT

During the concluding phase of therapy, peer supervision discussions centered on how to support patients in transitioning away from the OBT. Therapists noted that some patients, having reached their treatment goals, often felt no further need for the OBT. For others, however, the OBT had taken on a symbolic role in the therapeutic process, making its removal more sensitive. To ease this transition, some therapists introduced the idea of a transitional object, often drawing on tools from occupational therapy, such as a fidget ring. The choice of tool depended on the aspect of the OBT that patients valued most, whether it was the vibrotactile feedback, the act of carrying the instrument, or the sense of support it represented.

Patient interviews revealed that letting go of the OBT was experienced as a challenge, especially for those who had integrated it into their daily routines. Some described the use of the OBT as an embodied habit, noting that it took 2-3 weeks both to adopt and to unlearn after returning the instrument at the end of treatment. Others described a sense of emotional loss, particularly the perceived connection to their therapist that had been reinforced through the OBT’s responsive vibrotactile feedback. For some, these 2 aspects were intertwined: both the physical act of pressing the button and the emotional connection it symbolized were missed after the instrument was withdrawn.

One woman, for example, was given a rock with a symbolic meaning as a transitional object. In an interview, she shared that while the gesture was appreciated, she still missed the vibrotactile feedback from the OBT, which made her feel connected to her therapist. In addition, 2 patients expressed a strong desire to retain access to their data, emphasizing the valuable insights they gained from using the OBT.

From the therapists’ perspectives, discussed in peer supervision, these difficulties were partly attributed to the sudden absence of ongoing data collection and collaborative data analysis. These features of the OBT had supported both the therapeutic alliance and engagement throughout the treatment process, and their removal sometimes left a noticeable gap as therapy concluded.

## Discussion

### Principal Findings

Our study demonstrates how PAR methodology can be used to codevelop a psychotherapeutic treatment concept for refugees with CPTSD. Through iterative and collaborative processes involving therapists, patients, and researchers, we developed a culturally sensitive and personalized treatment approach in real-world clinical settings that integrates the OBT and the data visualization tool and can be used across psychotherapeutic modalities. Below, we discuss our findings about the 3 RQs that guided this study: ethical and practical considerations (RQ1), the setting and achievement of therapeutic goals (RQ3), and the interpretation and use of self-tracking data in therapy (RQ4).

### Ethical and Practical Considerations of Self-Tracking in Psychotherapy

Our findings on RQ1 highlight several ethical and practical considerations for integrating the OBT and the data visualization tool into psychotherapy with refugees. One particularly interesting insight from our study is the variability in how patients engaged with the OBT, both in terms of duration of OBT use, ranging from 22 to 366 days, as well as the number of daily observations, ranging from an average of 0.7 to 37. Most of this variability is likely to be explained by the inherent flexibility in tracking different target phenomena. However, the variability could also be partly due to some patients experiencing technical issues with the OBT, and not entirely unexpected with the OBT being a research prototype. For example, gaps in data collection were observed due to technical issues such as battery problems, running out of memory space, as well as practical issues such as misplaced instruments or therapists forgetting to return the OBT to the patient at the end of the session. Although patients were generally able to re-engage with the OBT, these gaps in data collection highlight the need for further technical and practical adjustments to ensure more consistent tracking. Addressing these issues could enhance the reliability of self-tracking data, thereby making it a more dependable tool for therapeutic interventions. These disruptions raise important practical questions about the workflow for self-tracking technology in psychotherapy, as periods without data could hinder the therapeutic process.

From an ethical perspective, the variability in how different patients responded to self-tracking highly underscores the relevance of personalizing self-tracking to the individual needs of patients. For some, particularly those tracking distressing phenomena, the act of self-tracking seemed to be overwhelming sometimes. One patient, who was experiencing emotional disturbances, found the OBT to intensify her feelings of being overwhelmed, leading her to discontinue its use. Additionally, that was even though she reported gaining insights into her maladaptive behavioral patterns. This raises ethical concerns about the potential burden that self-tracking can impose on vulnerable patients who are struggling with emotional instability. Given that refugees with complex trauma often face severe mental health challenges [7], it is essential to ask whether self-tracking adds to these burdens or merely brings existing vulnerabilities to light. Our findings suggest that self-tracking likely reveals pre-existing challenges, though further research is needed to substantiate this and examine how the self-tracking technology can be applied in diverse patient populations.

However, for other patients, the adaptability of the OBT and the flexibility in selecting target phenomena were a strength. The ability to shift the focus from self-tracking a distressing phenomenon to more positive, alternative coping strategies seemed to give some patients a sense of control and allowed them to use therapeutic interventions in their daily lives. This highlights the strength of the personalization enabled by the OBT, aligning with previous research that emphasizes the empowering effect of active patient participation in mental health care [41]. Nevertheless, this variability in the use of the OBT across different patients also presents a challenge, as the flexible application of the OBT can make it difficult to establish a consistent approach for its use in therapy. This aligns with findings from a study by Peñuela-O’Brien et al [11], who found that health professionals often adapt their practices to accommodate the diverse needs of refugee patients, leading to a range of therapeutic approaches. Future research should explore how to find a balance between the flexibility in use of the OBT and the value creation seen from a therapeutic perspective.

Lastly, the role of peer supervision was instrumental in helping therapists navigate these ethical and practical complexities. Peer discussions allowed therapists to reflect on the best strategies for introducing the self-tracking technology into therapy, balancing the potential benefits with the risks of cognitive overload. This highlights the importance of ongoing professional support when integrating new technology into therapy, particularly when working with vulnerable populations. For the treatment concept, we recommend incorporating regular peer supervision as part of the concept to ensure ethical and effective use of the technology in diverse clinical settings.

### Setting and Achieving Therapeutic Goals

Our findings reveal the complexities of using the OBT to align self-tracking activities with therapeutic goals, especially as the target phenomena shifted throughout therapy. This difficulty may stem from the novelty of using the OBT to track subjective experiences related to mental health challenges and integrating self-tracking data into therapy. One of the key challenges was the difficulty in operationalizing abstract emotional states such as “anxiety” and “anger” into discernible phenomena that could be recognized by the patient and tracked. As a result, some therapists and patients struggled to maintain consistent alignment between the self-tracking activity and the therapeutic goals, particularly when the phenomena being tracked changed frequently. This reflects the broader challenges faced in psychotherapy with refugees, where therapists often deviate from standard protocols to accommodate the cultural and complex needs of patients, but there is insufficient transparency in how this adaptation is carried out [42]. Integrating self-tracking technology introduces a new dimension to therapy, requiring both therapists and patients to learn and adapt. The early stage of the treatment concept development and the iterative nature of the PAR methodology likely contributed to a steep learning curve, which could have complicated the establishment of clear, consistent therapeutic goals.

Moreover, the flexibility of the OBT to track both distressing phenomena and alternative coping strategies created opportunities for patient engagement but also posed challenges in maintaining a clear therapeutic focus related to the goal of the therapy. For some patients, tracking alternative coping strategies such as BEs was more easily operationalized and helped foster a sense of control over their mental health. However, others who frequently changed their target phenomena experienced confusion about the tracking activity, and the relation of this to therapeutic goals was not always clear for either the therapist or the patient. This variability in the use of the OBT highlights the need for therapists to continuously reassess and adapt the target phenomena to ensure they remain relevant to the patient’s evolving therapeutic needs while maintaining a focus on the overall therapeutic goal.

At the same time, it is crucial to balance flexibility with consistency. Frequent changes to the target phenomena can interrupt the therapeutic process and confuse, making it more difficult for both patient and therapist to observe meaningful patterns and progress. Therefore, this study recommends limiting how often the target phenomena are changed and making shifts only when there is a clear therapeutic benefit. This method helps keep the therapeutic focus stable while still allowing for necessary adjustments. However, even when the target phenomena shifted frequently or the definitions became less clear, patients still gained insights into their mental health challenges, reinforcing the importance of collaborative goal-setting and self-tracking as an effective therapeutic instrument.

One possible explanation for why some treatment courses seem to have less focus on the overall therapeutic goal of the therapy, and instead an increased focus on the tracking activity itself, could be due to the design of the logbooks. Here, therapists were not explicitly asked to link tracking activities to specific therapeutic goals. This challenge was reinforced by the underuse of the hypothesis list in the logbooks, which may have contributed to a focus on tracking the phenomena themselves rather than linking them to broader therapeutic objectives. This suggests that further refinements in the design of the logbook and more structured guidance for therapists are needed to better connect the self-tracking activity to the overall therapeutic goal of the treatment.

Peer supervision sessions played a critical role in helping therapists navigate these challenges, providing room for supervision and reflection of the target phenomena and tracking protocols as therapy progressed. The iterative nature of the PAR methodology allowed therapists to collaboratively explore new approaches and refine the use of the OBT in line with patients’ needs, demonstrating the importance of ongoing professional support when integrating self-tracking technology into therapy. For the treatment concept, we recommend clarifying the therapeutic goal before adapting or changing the target phenomenon to ensure that the tracking activity remains aligned with the overall direction of therapy.

### Interpretation and Use of Self-Tracking Data

Our findings show that integrating self-tracking data into therapy sessions presents both opportunities and challenges for therapists and patients. The OBT’s potential to positively influence patient engagement and offer a feasible and personalized way to track mental health phenomena was a key insight. Many patients felt empowered by taking an active role in tracking their mental health, which aligns with existing literature showing that active patient involvement increases satisfaction and feelings of empowerment [41]. For instance, the long-term engagement observed in our study, with patients tracking for up to 366 days, suggests the broad applicability of the OBT across diverse patient groups, irrespective of age or cultural background.

However, integrating self-tracking data into therapy sessions presented challenges for some therapists. One notable issue was that, despite the collaborative analysis of data during sessions, therapists reported that it was sometimes difficult to balance the dual attention required to interpret the data while remaining present and attending to the patient’s immediate needs. This tension between the use of traditional therapeutic methods and the analysis of self-tracking data suggests that integrating digital health instruments into therapy may require more than simply adding data-driven components: it calls for a rethinking of how these tools interact with the flow of therapy. Therapists found that while the OBT provided valuable insights, it, unsurprisingly, required additional effort to manage alongside standard therapeutic practices.

We initially anticipated that the self-tracking data would lead to more systematic functional analysis, a core component of CBT, where specific behaviors and emotional triggers are mapped and addressed. However, in practice, some contextual cues associated with the data points were analyzed, but the comprehensive approach of functional analysis was not fully adopted. The self-tracking data served more as a navigational tool that helped therapists monitor patient engagement with the OBT and their target phenomena, as well as their overall progress. This finding aligns with previous research, which has noted that while digital tools can offer valuable data, the ability to integrate and analyze this data effectively in therapy remains a challenge [43]. It seems likely that clearer guidelines on how to integrate functional analysis with the self-tracking data provided by the OBT, as well as specific examples, might have facilitated more consistent adoption of this approach. The cognitive load of managing both the self-tracking data and the therapeutic conversation in-session, coupled with the developing nature of the treatment concept in this study, may also explain why comprehensive functional analyses were not fully used.

Notwithstanding these challenges, our findings also reveal the OBT’s potential to foster deeper patient engagement in therapy. By actively involving patients in the integration of the OBT and the self-tracking activity, the OBT empowered them to take ownership of their treatment, particularly through the tracking of personally meaningful phenomena. This aligns with the study by Kazantzis and Lampropoulos [44], who emphasize the importance of involving patients in the cocreation of therapeutic activities, such as homework, to enhance engagement and promote self-efficacy.

The added cognitive load of interpreting self-tracking data alongside ongoing therapeutic tasks might explain the underuse of certain tools in the logbook, such as the hypothesis list. To overcome this, future development work should aim to simplify and better integrate data analysis into routine therapeutic practices, allowing therapists to leverage self-tracking data more effectively to enhance the therapeutic process and patient outcomes.

Moreover, the vibrotactile feedback provided by the OBT upon button presses emerged as an unexpected but important therapeutic element. Some patients reported that the vibrotactile feedback from pressing the OBT button acted as a grounding technique, helping them manage distress in the moment. For these patients, the vibrotactile feedback created a sense of control over their symptoms, reflecting the findings from previous studies that users often form stronger connections with tangible, interactive devices such as jewelry, which can evoke more personal and intimate interactions compared to abstract digital interfaces [45]. This feature was also perceived as supporting nonverbal communication for some of the patients. By offering the patients a nonverbal means of expression, the OBT seemed to serve as a bridge between patient and therapist, allowing for deeper insights into the patient’s mental state without the need for explicit verbalization. This reinforces the complementary role of technology in supporting traditional therapeutic communication methods, which, as Woodward et al [46] emphasize, can enhance accessibility and engagement in mental health interventions, particularly in contexts where traditional communication may be challenging.

One of the most significant contributions of the OBT was its potential to strengthen the therapeutic alliance between patients and therapists. Our findings suggest that the OBT positively affected all 3 components of the therapeutic alliance model by Bordin [23]: bond, goals, and tasks. By engaging patients in defining target phenomena and collaboratively analyzing the data, the OBT fostered a deeper sense of partnership between therapist and patient. This collaborative approach helped clarify therapeutic goals, while the use of the OBT for tracking specific phenomena ensured that both patient and therapist were aligned on the tasks of therapy. This aligns with previous studies emphasizing the importance of the therapeutic alliance in treating refugees, particularly the need for mutual understanding and trust in addressing complex trauma [10]. Similarly, Mohl et al [47] reported that patients who perceived a poor therapeutic alliance were more likely to drop out of treatment. Strengthening the alliance in this way appears to be crucial for maintaining engagement and facilitating therapeutic progress, particularly for patients with complex trauma histories.

Interestingly, therapists in our study often viewed the OBT as particularly useful in supporting homework activities between sessions. The real-world application of the OBT allowed therapists to gain insights into how patients were engaging with therapeutic interventions outside of the therapy session. This proved particularly useful in addressing the challenges posed by language barriers, as the OBT allowed patients to engage with therapeutic tasks in a nonverbal, tangible way, bypassing the need for complex verbal explanations. The ability of the OBT to act as an instrument to support homework rather than as a primary object of analysis during therapy sessions also suggests that self-tracking data may be best used to supplement, rather than dominate, the therapy session.

### Limitations

Even with the challenges, this study delivered promising results. This study was conducted at DMTT, and while this is a strength in the PAR design, factors such as turnover of personnel, ongoing projects, and the decision to relocate the clinic during this study’s period may have impacted both the iterative process of developing the treatment concept and the onboarding of therapists to this study. These circumstances likely affected the consistency of data collection and could have influenced therapist engagement with using the self-tracking technology in therapy sessions.

The participating patients could have been beyond interviews involved in the PAR framework; however, due to their vulnerable status and need to protect their privacy, we chose to involve them indirectly. Furthermore, physiotherapists were not included in the cocreation process, even though it became evident during this study that many interventions were related to the patients’ physiotherapy treatments. Including physiotherapists could have enriched the cocreation process and provided more comprehensive insights into the treatment interventions.

There were technical issues related to the OBT’s battery life. The OBT depends on obtaining a GPS signal to set the clock in the event the battery becomes depleted. If the OBT was not charged and restarted near a window, it could not receive a GPS signal, in some situations, causing the battery to drain prematurely due to the retries of trying to acquire a GPS signal.

### Conclusions

This study sets out to codevelop and refine a self-tracking-assisted psychotherapeutic treatment concept for refugees with CPTSD. Building on a feasibility pilot study [17], we engaged patients, therapists, and researchers in a collaborative process to codevelop a treatment model that extends psychotherapy beyond the clinical setting.

Findings suggest that the OBT and the data visualization tool supported patient engagement by enabling in-the-moment self-tracking, creating agency, and strengthening the connection between daily experiences and therapy. Therapists reported that the self-tracking data provided detailed insights that enhanced personalization and informed clinical decision-making. The flexibility of the method allowed it to be adapted to individual needs and therapeutic goals.

By embedding lived experience into psychotherapy, this study shows the potential of momentary, low-burden self-tracking as a meaningful contribution to personalized care. Future research should explore this approach in other clinical contexts and assess how it can further support engagement, alliance, and continuity in care.

## Appendix

Multimedia Appendix 1 Worksheet on questions for OBT treatment. OBT: One Button Tracker.

## Abbreviations

ACS: alternative coping strategy
BE: breathing exercise
CBT: cognitive behavioral therapy
CPTSD: complex posttraumatic stress disorder
DMTT: Department of Multidisciplinary Trauma Treatment
HCI: human-computer interaction
ICD-11: International Statistical Classification of Diseases, Eleventh Revision
IT: intrusive thought
OBT: One Button Tracker
PAR: participatory action research
PTSD: posttraumatic stress disorder
RQ: research question

## Data Availability

The datasets generated and analyzed during this study are not publicly available due to the sensitivity of the participant data and the small cohort size. All participants were refugees undergoing psychotherapy for complex posttraumatic stress disorder, and consent covered the use of anonymized data for this specific study only. Researchers may contact the corresponding author to request access to selected deidentified materials, where feasible. Such requests will be considered on a case-by-case basis per Danish data protection legislation.
