# Supplementary material for: Collaborative Development of a Self-Tracking Assisted Psychotherapy Treatment Concept for Refugees With Complex Posttraumatic Stress Disorder: Participatory Action Research
Source: JMIR Form Res. 2025 Oct 7;9:e66663. doi: 10.2196/66663 (PMC12541264; doi:10.2196/66663)
Supplement: Multimedia Appendix 1 [file formative_v9i1e66663_app1.docx]

**Target phenomenon**

Inquire into specific actions or thoughts. The more precisely a symptom or phenomenon can be defined, the better the patient can observe it. Look for somatic markers and frequent occurrence (several times a day rather than a few times a week).

For example:

- Keep it **simple**: Focus on a single target phenomenon (at least to start with).
- **Frequency**: Choose a target phenomenon that occurs with some frequency. If it occurs only a few times a week, it may be difficult to track.
- The identified target phenomenon should present itself **clearly** in relation to what else is happening, so there is no doubt about its occurrence – and thus no doubt about whether to press the OBT or not.
  - If there is doubt whether a potential target phenomenon can be tracked or presents itself clearly enough, consider if there could be a "proxy phenomenon" for what you are interested in. That is, a phenomenon that occurs in connection with the phenomenon of interest and could be focused on as a "proxy."
  - For instance, there could be a somatic marker associated with the target phenomenon, which could then be tracked as an advantage.
- In some cases, tracking a given phenomenon may be undesirable or uncomfortable if it is associated with something negative. In such cases, consider whether you can "reverse tracking" and instead track something associated with something positive.
- Be ready to redefine the target phenomenon if it, for some reason, does not work out. It may be difficult to track, not present itself clearly, or be difficult in certain social situations.
- **Observe** first: Start by solely observing the occurrence of a target phenomenon. That is, start by creating an overview of the occurrence before possibly introducing an intervention.
- As a target phenomenon, one may choose an **intervention**. That is, tracking the performance of an intervention, such as a distraction exercise or similar. This way, an overview can be created of how often an exercise is actually performed.

**Hypothesis list:**

The hypothesis list should be relevant to the patient and not too ambitious. Use factual questions that can be compared against data when it comes in. The more context there is, the more the hypothesis can be held up against it.

The goal is not to have the "perfect" hypothesis but hypotheses that are good enough to support the process in continuing. Surprises that data show something other than what the hypotheses suggested can prove to be exceptionally productive. A hypothesis is formulated based on the best available knowledge at the given time and is therefore of a temporary nature.

Possible context to inquire about:

- **When** (time): Frequency - how often, time of the week, time of the day
  - For example: "Are there times when you experience this particularly much?" Or times when the target phenomenon occurs less frequently? Could there be patterns in relation to the time of day, weekdays, also differences between weekdays and weekends, months, or seasons?
- **Who:** Other people or social contexts.
  - For example: Does the target phenomenon occur more or less in certain situations and/or with specific people or groups/types of people? Are there activities, e.g., household chores, work, shopping, physical activity, etc., that are associated with more or fewer occurrences?
- **Where** (place): Places, environments, situations.
- **What**: Other triggering factors.
  - Places and environments: What is the occurrence of the target phenomenon in relation to staying in different places or in environments with different characteristics, e.g., noise/silence, light/dark, cold/warm, etc.?

**Observation protocol:**

The observation protocol describes:

1. Which target phenomenon is being tracked
2. How to use OBT for tracking the given phenomena (press)
3. Practical aspects of using OBT in daily life

In the observation protocol, describe as specifically as possible:

- Where do you keep the tracker? How do you ensure to always carry it with you?
- How do you press the tracker when the target phenomenon occurs? (Initially, one short press)
- More than one press for multiple target phenomena:
  - In a multi-press protocol, specify how to press for each target phenomenon.
  - For example, 1 press for target phenomenon A, 2 presses for target phenomenon B.
  - For example, a *short* press for target phenomenon A and a *long press* for target phenomenon B.
- Observation and intervention.
  - For example, one press when the target phenomenon is observed and two presses when the intervention is performed.
